# Impact of stewardship pharmacist driven MRSA nasal surveillance and de-escalation of anti-MRSA therapy (STEW PHARM MRSA NASAL SURVEILLANCE)

**DOI:** 10.1017/ash.2024.443

**Published:** 2024-12-12

**Authors:** Jessica Dillon, Domenic Vita, Jessica Abrantes-Figueiredo, Dora Wiskirchen

**Affiliations:** 1 Department of Pharmacy, Saint Francis Hospital and Medical Center, Hartford, CT, USA; 2 Department of Infectious Disease, Saint Francis Hospital and Medical Center, Hartford, CT, USA

## Abstract

**Objective:**

To determine if implementing stewardship pharmacist-driven methicillin-resistant *Staphylococcus aureus* (MRSA) nasal surveillance increases use of the test and reduces the inappropriate use of vancomycin for MRSA coverage in patients with pneumonia.

**Design:**

Retrospective pre-/post-intervention study.

**Setting:**

Large teaching acute care hospital.

**Participants:**

Adult patients receiving vancomycin therapy for treatment of pneumonia.

**Methods:**

A stewardship pharmacist ran a report of admitted patients receiving vancomycin and reviewed the patients’ records. If the patient’s indication was pneumonia and a MRSA nasal swab had not been ordered, the pharmacist contacted the patient’s provider and requested an order for it. Upon receipt of a negative MRSA nasal swab result, the pharmacist recommended discontinuation of vancomycin if appropriate.

The control group was four weeks prior to the stewardship intervention, where there was no dedicated stewardship pharmacist reviewing MRSA swab utilization. The primary outcome was percentage of patients who had a MRSA swab ordered. Secondary outcomes included percentage of patients who had vancomycin appropriately de-escalated based on MRSA nasal swab results and length of vancomycin therapy.

**Result:**

Percentage of swabs ordered increased from 36.1% (22/61) to 83.7% (41/49) with pharmacist intervention (*P* < 0.0001). The rate of vancomycin de-escalation following a negative MRSA swab increased from 19.7% (12/61) to 61.2% (30/49) with pharmacist intervention (*P* < 0.0001).

**Conclusion:**

The results suggest implementing a pharmacist driven MRSA nasal surveillance program into practice could increase the number of MRSA nasal swabs ordered and promote timely de-escalation of vancomycin in patients with pneumonia.

## Introduction

Methicillin-resistant *Staphylococcus aureus* nasal surveillance (MRSA swabs) has been highlighted in recent literature and the *2019 IDSA Pneumonia Treatment Guidelines* as a tool to avoid unnecessary empiric coverage for MRSA in pneumonia.^1,2^ A MRSA swab has a 96.5% negative predictive value can be utilized by clinicians to de-escalate intravenous vancomycin and other anti-MRSA therapies for empiric therapy of community acquired pneumonia (CAP) and hospital-acquired pneumonia (HAP).^1,2^ De-escalation of vancomycin for pneumonia when MRSA swabs are negative has been shown to reduce the duration of anti-MRSA therapy without increased in-hospital mortality or hospital length of stay.^3,4^ There is currently no role for positive MRSA swabs in adding therapy for pneumonia given the low positive predictive value of 44.8%.^1^

Pharmacists are in an ideal position to be monitoring MRSA swab surveillance. Part of the daily workflow of the pharmacist includes daily chart review to ensure proper antibiotic use based off indications and microbiology cultures. By allowing pharmacists to drive the MRSA nasal surveillance process, this would maximize the workflow of the pharmacist and relieve tasks from the providing physicians. Pharmacist driven MRSA nasal surveillance has the potential to reduce unnecessary MRSA coverage and reduce complications associated with inappropriate antibiotic therapy such as risk of adverse drug events, development of antimicrobial resistance, prevent drug-drug interactions, and reduce hospital expense. This study’s purpose is to measure the impact of a pharmacist conducting MRSA nasal surveillance and vancomycin de-escalation.

## Methods

This study is a retrospective pre-/post-intervention study approved by the Trinity Health Of New England institutional review board. This study evaluated the effectiveness of the stewardship initiative for MRSA swabs/vancomycin de-escalation. The antimicrobial stewardship team completed a four-week initiative from April 10, 2023, to April 28, 2023, to identify all patients actively being treated with vancomycin for pneumonia that would benefit from MRSA swabs and de-escalation of vancomycin. This period was compared to a control period, March 13th, 2023, to April 7th, 2023, the four weeks before the initiative started. During the control group, which followed the standard of care at our institution, each unit had an assigned floor pharmacists who occasionally monitored MRSA swabs and made recommendations to providers as time allowed outside their other daily responsibilities.

In the intervention group, a daily list of patients on vancomycin was generated from the electronic health record (EPIC). The stewardship pharmacist reviewed the electronic health records of each patient identified to determine the indication for vancomycin. If the indication was pneumonia, the pharmacist would make recommendations relative to the MRSA swab as necessary. Providers were contacted and asked to order a MRSA swab if a patient was on vancomycin for pneumonia and had not had one ordered or performed within the past 7 days. All patients with MRSA swabs were followed and if negative providers were contacted by the stewardship pharmacist and recommended de-escalation if appropriate to do so based off the swab and other microbiology cultures. If a patient had vancomycin therapy that crossed the control and intervention timeline, they were included in the intervention group only if the pharmacist intervened prior to discontinuation of vancomycin.

### Inclusion and exclusion criteria

Patients were included if they were adults younger than 89 admitted as inpatients and receiving vancomycin for community acquired or hospital acquired pneumonia. Those 90 years and older were excluded per institutional IRB protocol to protect patient information. Patients could be counted twice if vancomycin was discontinued and restarted for a new respiratory infection of interest. They were excluded if they were diagnosed with VAP or had confirmed MRSA pneumonia.

### Outcomes

The primary outcome was percentage of patients empirically treated with vancomycin for pneumonia who had a MRSA swab ordered at the beginning of vancomycin therapy. Beginning of therapy was defined as the first 24 hours of therapy. The exception was if a MRSA swab was ordered on a Monday for vancomycin started over the weekend in the intervention group. There was no stewardship pharmacist on Saturday or Sunday, so Monday was the earliest they could intervene. If the provider accepted the intervention that Monday, it would still be considered beginning of therapy.

Secondary outcomes included percentage of patients who had vancomycin appropriately discontinued following a negative MRSA swab; percentage of patients that had vancomycin inappropriately continued following a negative MRSA swab; average length of vancomycin therapy; and potential cost savings. If vancomycin therapy was given for multiple indications, all days of therapy were counted. The decision to collect data this way was made because there often was not clear documentation in providers’ notes when pneumonia was no longer a concern, and vancomycin was only being used for the other non-respiratory indication. The exception would be if there was a clear gap in vancomycin therapy for two different indications, specifically a respiratory infection of interest and a non-respiratory infection of interest. For example, if a patient was started on vancomycin for pneumonia, had it discontinued for multiple days, and was restarted for cellulitis, only the first course of vancomycin would be included in data analysis. If vancomycin was discontinued then restarted for pneumonia, both courses would be counted, excluding the days the order was discontinued. If a patient was on an extended dosing interval (i.e., every 48 hours) the days between doses counted towards days of therapy.

A cost analysis of potential savings was conducted utilizing wholesaler prices obtained by the pharmacy purchasing team and laboratory staff. The current defined daily dose of vancomycin is 2 grams.^5^ Over the course of our eight-week study, we looked at 110 patients, which when extrapolated would be 715 patients over the course of a year. At our institution, we purchase 10-gram vials of vancomycin that require reconstitution by a pharmacy technician. Each vial cost $18.99 when bought at a group purchasing organization (GPO) rate.

### Data analysis

This study included a comparator group. The comparator group was the four weeks prior to the implemented stewardship initiative. Based on our clinical and stewardship data to date, we anticipated a 40% event rate in the pre-initiative group and a 90% event rate in the post-initiative group. Assuming an alpha of 0.05 and power of 0.8, the study required a sample size of at least 13 in each group. The collected data was utilized to determine the frequencies of the primary and secondary outcomes within the study period. Primary and secondary outcomes were compared between the two groups using a Chi-square test, except for the secondary outcome of days of vancomycin therapy, which were compared using a t-test.

## Results

131 patients were screened for the study and 110 were included in statistical analysis (Table 1). For the primary outcome, we found a statistically significant difference favoring the utilization of a pharmacist driven stewardship initiative (Table 2). In the control group, only 36.1% (22/61) of patients had a MRSA swab ordered at the beginning of therapy; that number rose to 83.7% (41/49) in our intervention group (*P* < 0.0001). For our secondary outcomes, appropriate discontinuation of vancomycin was also statistically significant, with 61.2% (30/49) of all patients in the intervention group having his or her vancomycin discontinued following a negative swab compared to 19.7% (12/61) in the control group (*P* < 0.0001). When only looking at patients who had a MRSA swab ordered, 54.5% (12/22) and 73% (30/41) of patients with a swab ordered in the control group and intervention group respectively had their vancomycin discontinued following a negative result. Inappropriate continuation of vancomycin was statistically significant, favoring the control group. 12.2% (6/49) of all patients in the intervention group had vancomycin inappropriately continued, compared to 1.6% (1/61) in the control group (*P* = 0.0235). When looking at just patients with a swab ordered 4.5% (1/22) patients in the control group and 14.6% (6/41) patients in the intervention group had vancomycin inappropriately continued.

**Table 1.** Summary of patient screening in each group

	Control	Intervention
Total screened	73	58
Excluded for age	5	3
Excluded for VAP	3	3
Excluded for receiving treatment at outside facility	1	0
Excluded for confirmed MRSA pneumonia	3	3
Included in analysis	61	49

**Table 2.** Primary and secondary outcomes in control and intervention group

	Control (n = 61)	Intervention (n = 49)	*P* Value
**Primary Outcome**			
MRSA Swab ordered at the beginning of therapy, %(n)	36.1% (22)	83.7% (41)	<0.0001
**Secondary Outcome**			
Appropriately discontinued vancomycin following negative MRSA swab, %(n)	19.7% (12)	61.2% (30)	<0.0001
Inappropriate continuation of vancomycin following negative MRSA swab, %(n)	1.6% (1)	12.2% (6)	0.0235
Mean days of vancomycin therapy	3.85	3.53	0.32

The number of days of vancomycin therapy was not statistically significant between the two groups (3.85 vs 3.53, *P* = 0.32). However, if the length of therapy was reduced by one day, it would be estimated our site would save approximately $2,715.57 on drug cost annually. The projected savings do not include labor costs associated with reconstitution by a technician. In addition, by reducing the length of therapy, it would reduce the number of vancomycin levels needed to be run. The current cost for a vancomycin level at our institution is $87. Robust pharmacoeconomic studies are needed to fully ascertain the cost savings potential of utilizing MRSA swabs to de-escalate vancomycin, including but not limited to savings in vancomycin expenditures, pharmacy and nursing labor for drug preparation and administration, as well as vancomycin levels for therapeutic drug monitoring.

## Discussion

We compared the rate of MRSA swabs ordered at our institution when there was a dedicated pharmacist managing MRSA swabs and when there was not. Significantly more MRSA swabs were ordered when a pharmacist intervened, and more patients had vancomycin therapy discontinued. This process is in line with current IDSA guidelines that highlight the usefulness of MRSA swabs to de-escalate MRSA coverage.^1^

In the intervention group, there were statistically more patients who had vancomycin inappropriately continued, not due to inappropriate recommendations from the pharmacists, but rather because the increased number of MRSA swabs highlighted inappropriate practice. MRSA swab results can be utilized by antimicrobial stewardship committees to ensure providers are de-escalating therapy as necessary and provide education and interventions as necessary.

We found the days of vancomycin therapy was not statistically different between the two groups. The potential reason for this is multifactorial. Our institution utilized MRSA culture at the time of the study, which take one to three days to result.^4^ The amount of time to obtain results is significantly longer than the current gold standard PCR MRSA swabs, which can result in as quick at 30 minutes.^4,6^ During the study, swabs took an average of 32.1 hours from collection time to result, delaying negative results and vancomycin de-escalation. Other similar trials that utilized PCR tests found days of vancomycin therapy was significantly less in the pharmacist intervention groups.^7,8^ In addition, antimicrobial stewardship at our site was strong prior to our initiative. Our providers are trained to reevaluate empiric antibiotic therapy after several days, with many primary teams already de-escalating vancomycin early in therapy regardless of whether a MRSA swab was conducted.

Cost savings due to a pharmacist driven stewardship program would be maximized at facilities that currently use PCR MRSA swabs. Similar studies that looked at the implementation of a pharmacist driven MRSA nasal swab policy found reductions of vancomycin anywhere from 14.5 hours to 46.6 hours.^6–10^

The study had limitations. The rate at which MRSA swabs were collected varied. MRSA swabs are collected by nurses, and sometimes orders would go uncollected for days even after pharmacist intervention. Two patients in the control group required their MRSA swabs to be cancelled and reordered to prompt the nurse to collect it. Despite multiple days of vancomycin therapy, sensitivities of the MRSA swab should not be greatly impacted, but more research is needed on the subject.^3^ In addition, it is possible there were patients on vancomycin that were not intervened on because only one pharmacist was involved in the intervention who was not scheduled to work on the weekends during the intervention period. Therefore, if an order was started and discontinued between Friday night and Monday morning, the pharmacist would not see the order. However, we estimate this would be a small number of patients. All patients that were started on vancomycin over the weekend were intervened on Monday morning if they were still receiving treatment.

The results from this study can be used to support the wide-spread use of pharmacist driven MRSA nasal surveillance protocols at other institutions. At our site, we are implementing a pharmacist driven MRSA swab protocol, which will allow pharmacists to order MRSA swabs per protocol if the patient is receiving vancomycin for pneumonia. Our lab recently switched to PCR swabs, so it is anticipated this switch and implementation of the new protocol will allow for less broad-spectrum antibiotic use, shorter vancomycin therapy, and decreased drug and monitoring costs. This study may lead to future studies to evaluate the other downstream effects such as patient clinical outcomes, reduced vancomycin use, hospital cost, adverse drug events, drug-drug interactions, and antibiotic resistance.
